# On-site communication measures as a tool in outdoor recreation management: a systematic map

**DOI:** 10.1186/s13750-023-00305-2

**Published:** 2023-07-22

**Authors:** Sofie Kjendlie Selvaag, Rose Keller, Øystein Aas, Vegard Gundersen, Frode Thomassen Singsaas

**Affiliations:** 1 Norwegian Institute for Nature Research (NINA), Høgskoleringen 9, 7034 Trondheim, Norway; 2 Faculty for Environment and Natural Resource Management, Norwegian University of Life Sciences (NMBU), 1432 Ås, Norway

**Keywords:** Behavior psychology, Persuasion, Visitor management, Communication theory, Conservation messaging, Conservation psychology

## Abstract

**Background:**

Communication is a central tool used to manage the balance between outdoor recreation and environmental protection in natural areas. Several studies have evaluated different communication measures in case studies, but rarely are these measures compared across contexts. We review the literature guided by the question: what is the scope of evidence of on-site communication to change human behavior towards a more sustainable direction in outdoor recreation? Taking natural areas as our starting point, we map research-based distribution and abundance of communication measures, with emphasis on their outcomes and study design.

**Methods:**

The target population for our mapping review are outdoor recreationists and nature-based tourists who visit natural or near-natural settings. We examined studies that have crafted written, oral and visual intervention measures to change behavior by using persuasion, education and information rather than legal restrictions or bans. Examples of challenges addressed with communication measures include proper waste disposal, using designated trails, minimizing wear and tear at campsites, avoid disturbing wildlife, and encouraging appropriate and safe behavior. We did not restrict our search geographically. We searched publication databases for peer-reviewed published articles (WoS, Scopus) and forward and backward citation chasing. To identify grey literature we used the database IRMA and internet searches in Google Scholar supplemented with specialist searches. Inclusion criteria and related search terms were based on PICO and included population (P: people participating in outdoor recreation in natural settings), terms that denoted intervention (I: on-site communication measures in situ vs. C: no communication measures) and terms that denoted outcome (O: changed behavior). We screened first by title and abstract and finally full text. For each article selected for full-text screening, metadata was extracted on key variables of interest such as behavior category, context, targeted population, study design and outcomes.

**Review findings:**

Overall, we identified 54 studies that were assessed in the review. Our review documents growing academic interest looking at actual behavior change in outdoor recreation. Theory is often subsidiary to attempted behavioral change via communication and different situational aspects, such as targeted visitor populations and environmental context, as well as psychological factors remain underexplored in the literature. The primary communication medium in the reviewed papers is passive use of signs. Awareness raising is the dominant communication mode, but other modes such as emotions, identity, and social norms are common. The geographic distribution of the studies is highly skewed to the United States.

**Conclusions:**

The amassed studies have an uneven focus on different settings and mediums used to change behavior. Research could benefit from investigating different contexts and the state of the natural environment before and after interventions. We advocate for casting a wider disciplinary net and interest in qualitative investigations to produce data-rich studies of where and how sustainable behavior is encouraged and eventually achieved. Collectively, different disciplinary perspectives are required to understand the aspects that contribute to sustainable, and sustained, behavior change. It is important to distinguish what aspects of behavior change could be generalized across settings, and which purely contextual aspects drive behavior change.

**Supplementary Information:**

The online version contains supplementary material available at 10.1186/s13750-023-00305-2.

## Background

Nature-based tourism has grown rapidly in recent decades and environmental managers around the world are faced with a conundrum of facilitating quality nature experiences for visitors while limiting damage from high use and unsustainable behaviors (see e.g., [1]). Communication is social interaction through verbal information and nonverbal symbols where people exchange thoughts, messages or information [2, 3]. In nature-based tourism and visitor management in protected and recreational areas, managers have often turned to the communication process of interpretation or persuasion as a key tool in striking a balance between nature-based tourism growth and environmental protection [1, 4, 5]. There are four common approaches that often guide visitor management: limiting use, increasing supply spatially or temporally, reducing the impact of use and increasing the durability of the resource [5]. Passive communication measures such as signage and interpretive texts or active communication such as the presence of rangers can reduce the impact of use by guiding visitors towards sustainable behavior, and are in general preferred by visitors, managers and decision-makers, instead of management strategies that prohibit or restrict use of an area [5]. Communication to change behavior is vital for conservation policy [1, 6]. Whereas systematic reviews go into depth of a particular method or outcome, systematic maps are helpful to get a sense of the major advances and gaps in the literature. The aim of this map is to widely share existing knowledge of study design and communication strategies managers and researchers utilize to examine sustainable behavior change.

Scholars have for a long time been interested in measuring the effectiveness of a variety of communication measures used to manage recreation in natural settings (e.g., [7–10]). Different kinds of communication measures are not often compared (our preliminary search did not identify any existing cross-comparative reviews regarding behavior change), thus effectiveness of interventions is challenging to ascertain. Moreover, existing literature often addresses only singular aspects of communication theory and outcomes in visitor satisfaction, like changes in knowledge or attitudes instead of behavioral change [11–14]. Two notable exceptions are outlined in literature reviews by Kidd et al. [6] where they focused on conservation messaging and raising awareness or intended behavioral change, and by Esfandiar et al. [15] where they addressed general, pro-environmental behaviors or behavioral change in protected areas. However, these reviews have limited scope in looking at behavior change and do not address natural areas in general.

A systematic mapping that addresses studies’ methodological designs and context together with actual *measured* behavioral change is needed to advance our knowledge of how behavioral change towards more sustainable practices has been achieved through communication measures. One major line of theory in the literature has examined behaviors as rational and moral processes (e.g., value-attitude-belief, norms and theory of planned behavior, [15]), while the other has examined the design and delivery of persuasive communication and how individuals interpret content (e.g., elaboration likelihood model, [6]). As stated earlier, most studies look at self-reported behavior or behavioral intention, but actual behavior is also influenced by emotions, environmental features, and other contextual factors. This can lead to a different behavioral outcome than intended (i.e., a behavioral intention gap, [16]). Human behavior in nature is highly context dependent and it is important to be aware that a communication measure that works in one place and at one time may not work another place or in another time [17–20]. Many factors influence behavior, for instance the same measure might affect behavior differently among people with different demographic characteristics, or affect individuals differently depending on context, e.g., different types of trips (family day hikes, vs. wilderness adventures) and throughout an individual’s lifespan. Yet, due to established frameworks for understanding and analyzing human behavior we could identify some general findings about the settings and management guidelines that can help in the design of future communication measures.

### Stakeholder engagement

Our stakeholders helped define knowledge gaps and the research questions for the present systematic map (see the review’s protocol for further details; [21]). The stakeholders helped with the framing of the review, not in the process or production of the present map. During our search for articles, we reached out to expert contacts during two professional meetings to find master and Ph.D. theses, reports and articles not indexed in the search databases used. This was done through our networks in IASNR (The International Association for Society and Natural Resources) and MMV (The International Conference on Monitoring and Management of Visitors in Recreational and Protected Areas). Whomever attended and felt they had studies to contribute in our search for articles could send us emails with studies we later screened. The IASNR and MMV meetings had 10–32 individuals in our research area, and of these we received studies from six people.

## Objective of the review

### Primary question

- What is the evidence base of on-site communication in outdoor recreation to change human behavior towards a more sustainable direction in natural settings?
  - P: people participating in outdoor recreation,
  - I: on-site communication measures (in situ),
  - C: no communication measures,
  - O: changed behavior.

### Secondary questions

- Which theories and conceptual frameworks have been used to guide empirical studies on the effects of communication in guiding human behavior?
- What types of research design and methods have been used to evaluate the effectiveness of on-site communication measures?
- What type of unsustainable or unsafe human behavior has been addressed with on-site communication in visitor management?
- Which on-site communication measures have been studied?

## Methods

### Deviations from the protocol

We did not deviate from our original protocol [21] except in our screening process. The screening in Sysrev was more time-consuming than expected because it took time to come to agreements on inclusions. As stated in our original protocol, we discussed the inconsistencies and commonalities among reviewers’ for the first 25 articles in order to come to an agreement on the inclusion process. Due to the large number of articles and the strict inclusion criteria, we decided that each study should be reviewed by two reviewers, and we followed up with regular discussions regarding all inconsistencies. After 3000 articles (half of our sample), we were satisfied with our consistency and flow. Each reviewer proceeded to screen a portion (800 each) of the remaining articles alone. The full text screening proceeded with two reviewers instead of four as stated in the protocol since all four reviewers were consistent in selection and quadrupled reviews were superfluous.

Most importantly, some of our initial benchmark articles were not used in our final review as our screening discussions led to a more conservative interpretation of our inclusion criteria. We decided to focus on studies that examined observed behavior, rather than intended behavior, in order to contribute to the ongoing debates in the literature surrounding the intention-behavior and attitude-behavior gap [16].

### Search for articles

Search terms describing the population (people participating in outdoor recreation in natural settings) were combined with terms describing intervention (communication measures) and terms describing outcome (changed behavior). See Additional file 2: Supplement A for the search dates and the search strings used for each search conducted. All supplementary information is organized in separate sheets in Additional file 2. All search terms were in English. We searched Scopus and Web of Science bibliographic databases. Databases searched from WoS Core Collection were:

Science Citation Index Expanded (SCI-EXPANDED): 1987–present.

Social Sciences Citation Index (SSCI): 1987–present.

Arts & Humanities Citation Index (AHCI): 1987–present.

Emerging Sources Citation Index (ESCI): 2015–present.

Our initial search in Scopus and WoS returned a disproportionately high amount of medical studies, thus we identified signifiers of unsuitable health articles in order to remove them before uploading them into Sysrev for full screening (see description in last paragraph of this section). In addition, we conducted forward and backward citation searching (as defined in Haddaway et al. [22]). We included in our backward citation process our benchmark articles, especially because some of our benchmark articles were too old to be in found in Scopus and Web of Science.

We searched for grey literature studies adapting the same search strings via Google Scholar based on the title of the review. The search options were ‘any time’ and ‘any type’, ‘not include patents or citations’ and ‘sort by relevance’. The individual searches retrieved between 16,900 and 71,800 results, thus ‘sort by relevance’ was used to limit the number of search results considered. Google Scholar displayed 10 search results per page and when no relevant study was detected on a page based on the title and text related to the search terms the search stopped and no studies were included after this page. We used a specialist natural resource report website of U.S. public and protected lands (Integrated Resource Management Applications, IRMA) to find studies conducted within the U.S. because a substantial work on this topic have been conducted in the context of U.S. public and protected lands. The returns in Google Scholar and IRMA were small compared to Scopus and WoS, so we removed unsuitable articles from Google Scholar and IRMA before the full screening in Sysrev. As a last step to ensure that we obtained all relevant literature we contacted external experts for input.

After several deduplication rounds (both automatic and manual), our EndNote library contained 8561 papers. We found many medical studies in our database and decided to exclude all papers where journal title included at least one of the following words: health, medicine, neuro, clinic, nursing, transport, toxic, surgery, parkinson, pharmacolog, disease, cell, surgic, cancer, chemist, alzheimer, pediatr, dermatolog, dement, brain, medica, disorder, drug, aging, nurse or molecul. This was done through the search function in EndNote. This resulted in the removal of 2013 papers from our database. Nevertheless, we still had a substantial number of publications about “Parkinson” or “parking” and decided to remove all publications that had one or two of these words mentioned in the title or abstract. After this was done, we began the screening process in Sysrev.

### Article screening and study eligibility criteria

#### Screening process

We conducted an initial screening of articles in Google Scholar and IRMA before our main screening in Sysrev based on the eligibility criteria at the title and abstract level. After the initial exclusion process, the results were uploaded to a shared project on Sysrev for screening. The reviewers proceeded with screening by title and abstract, followed by full text. The title and abstract screening were done together by two independent reviewers. The first 3000 studies were reviewed in this way, with consistent group check (4 reviewers in total, two groups of two). We did not conduct a statistical test of consistency, but rather checked for consistency through inter-reviewer discussions about reasons for inclusion/exclusion of articles. Consistent agreement was determined when independent reviewers reported the same eligibility criteria based on PICO to justify the outcome of their decision. In the case of a single study having multiple records (e.g., a report and a journal article), we selected the longer and most complete record. After a number of group checks we reached consistent agreement, therefore the last 3000 studies were equally divided among the reviewers for independent review. After the screening by title and abstract was completed, the first 10 full text reviews were screened by two reviewers independently. Agreement between the reviewers was 100%, thus full text screening proceeded separately by the two reviewers working on different studies. The final included studies were coded together by the two reviewers. The two reviewers coded the studies in the same room and examined their coding after each study. In case any inconsistencies were found, they discussed and resolved them immediately. Replicability of eligibility decisions was measured and reported and excluded articles (and reasons for exclusion), are provided in Additional file 2: Supplement C. None of the articles reviewed were authored by any of the four reviewers.

We mapped only studies on communication measures that clearly have defined on-site or other experimental setting intended to influence behavior at that specific time, and we also excluded studies that delivered communication before visitation (e.g., via internet and printed material such as newspapers and books). Our review had a specific focus on natural or near-natural areas, though no geographical bounds, and did not address behavior where people knowingly had engaged in illegal behavior or vandalism. Our review describes theories and management frameworks that have been the basis for designing these measures. The review identified knowledge gaps and other challenges communication-behavior studies often face. For an extensive description of the design and conduct see the protocol made for this systematic map [21].

#### Eligibility criteria

Population: People participating in outdoor recreation as long the activities take place in natural areas which are not heavy facilitated. Examples of natural areas: forests, mountain areas, bushlands and lakes/rivers. Study areas might include any geographic region globally.

Intervention: Any implemented communication measure, both written/printed, visual and oral/audio where the wanted outcome is to encourage pro-environmental behavior. In our review, pro-environmental behavior includes proper waste disposal, staying on designated trails, minimizing campfire impacts, respecting and not feeding wildlife, fee and regulation compliance, showing consideration towards other visitors and following safety measures.

Comparator: No communication measure at the same place, but at a different time or in a similar setting or testing the effect of different communication measures at the same place or in similar settings.

Outcome: Changed behavior including both wanted and unwanted behavior based on how it is affecting the environment or people.

Study type: Any primary empirical research study, both observational studies and experimental/intervention studies published as reports or articles.

#### Study validity assessment

We did not formally appraise the validity of included studies. Our review briefly describes the design of each study (i.e., study design, theoretical framework, measurements, target population, sample), and compares themes across studies in Additional file 2: Supplement D. Our strict inclusion criteria limited study inclusion to those that had actual, observed behaviors in real life settings, avoiding intended or self-reported behavior. Additional file 2: Supplement D can also be used as a guide to find appropriate studies describing relevant themes for further research.

#### Data coding strategy

All studies that passed the eligibility criteria were mapped and coded guided by the secondary research questions. The meta data and themes considered in our review are listed in the protocol [21] and Additional file 2: Supplement D. The list from the protocol was compiled using *Communication research in outdoor recreation and natural resources management* [17], *Influencing human behavior* [19], *Navigating Environmental Attitudes* [18], *Promoting persuasion in protected areas* [23] and *Social Science Theory for Environmental Sustainability* [20]. These books and reports guided and helped identify relevant themes for our review. The list of extracted data records is presented in Additional file 2: Supplement D. In addition, reported behavior change and the authors’ stated reasons for unaltered behaviors can be provided upon request.

#### Data mapping method

The presentation of the collected studies and the data they contain relies primarily on the extracted data records. The extracted data consists of text and coding as the map is focusing on a wide range of questions. The studies were organized by behavior category, context, targeted population, study design and outcomes. The protocol [21] displayed how the relevant literature is organized and descriptive statistics regarding the distribution of the articles is provided in review findings. Clusters were used to explore relationships within and between studies to identify key knowledge gaps, knowledge clusters and locate characteristics of the studies that assess the effectiveness of on-site communication measures to change human behavior in outdoor recreation.

## Review findings

### Review descriptive statistics

**Fig. 1:**
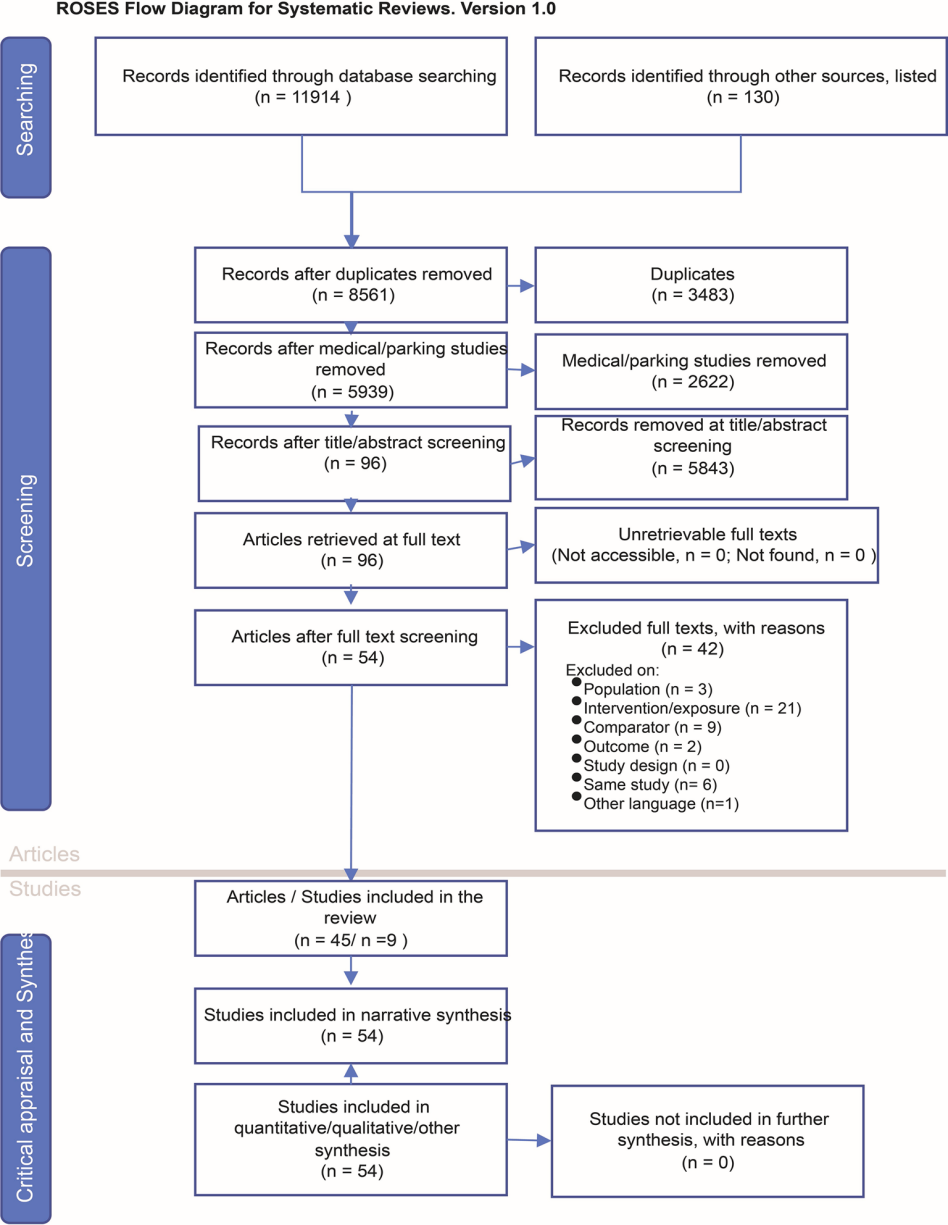
The data mapping method used for this systematic map and the number of sources at each stage (n) (Source: [45])

Through the database searches 11,914 studies were identified (WoS 4370 studies, Scopus 6841 studies and Citation Chaser 703 studies) and 130 studies (Google Scholar 92 studies, IRMA 13 studies and specialist contacts 25 studies) from the grey literature searches (Fig. 1). Of these, 29% (3483 studies) were duplicates leaving 8561 studies. After removal of 2622 irrelevant articles, we had 5939 studies to screen in Sysrev at the title and abstract level. The screening process resulted in an inclusion rate of 1.6%, a total of 5843 records removed, leaving us with 96 articles to screen at full text stage. The full text screening removed 43% of the 96 papers in the remaining pool and the review consequently identified 54 studies that were included in the review and synthesis. Many were removed due to outcomes not in line with inclusion criteria (e.g., looking at self-reported behavior or behavior intentions) or replication in multiple forms (e.g., report and journal article from the same study). See Additional file 2: Supplement C for description. Two articles we included were experimental in scope and game-based presenting dilemmas to respondents. Even though these studies did not look at actual behavior, they were designed to activate a specific context dependent behavior rather than intentions often measured in survey-based designs. In Additional file 2: Supplement C, the primary reason for exclusion is listed for each source, however there were often several reasons for exclusion at full-text level. The most common was that the intervention criterium was not met. We included all studies that met our eligibility criteria and did not conduct an appraisal of the validity of included studies. In Additional file 2: Supplement D, the full list of the 54 included studies is provided along with a summary of extracted data records.

**Fig. 2:**
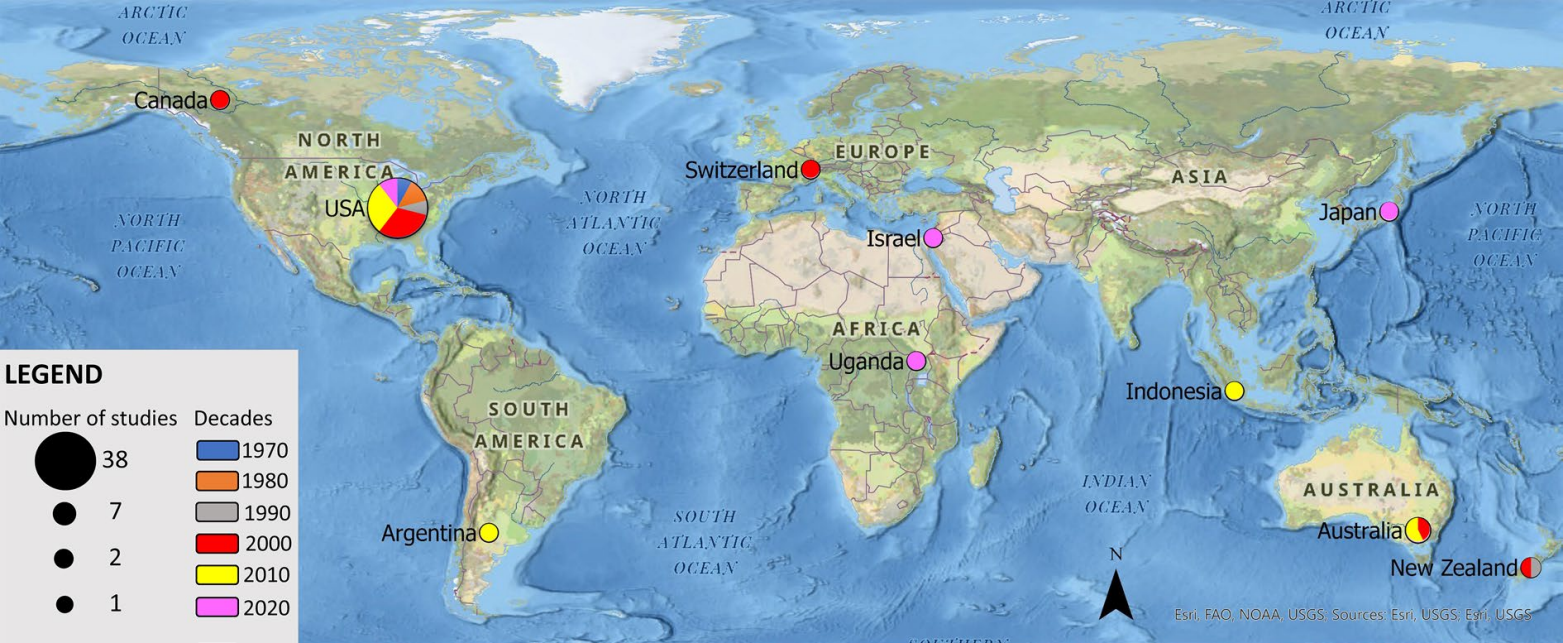
Publication decade and number of studies for each country investigating communication measures to change behavioral towards a more sustainable direction in natural settings

Most articles concerning communication measures to address visitor behavior appeared starting in the 2000s (Fig. 2). The oldest article identified was from 1969, but only 12 articles met our eligibility criteria in the decades up to 2000. Our included 54 articles spanned 27 different journals. The dominant publication sources were the Journal of Interpretation Research (6 studies) and Journal of Park and Recreation Administration (4 studies). Most of the journals with relevant articles were about tourism, recreation (16 studies) or education/interpretation (12 studies), with some appearing in conservation and environmental management journals (5 studies). From grey literature we included nine theses, dissertations and official reports from land management agencies. The geographical distribution of study locations was primarily the U.S. (70%, n = 38), followed by Australia (13%, n = 7). North America (US/CA) and Oceania (AT/NZ) represented almost 90% of the studies (n = 48, Fig. 2).

### History of the field

Before 1999, the US was the only country represented in the literature. Espiner [24] then in 1999 published a study on the effectiveness of both existing and introduced hazard warning signs to glaciers on visitor behavior in New Zealand, and 6 years later a study from Australia evaluated safety signs about the dangers of diving or jumping into natural watercourses [25]. This was also the first study that looked at visitor behavior in ocean/freshwater systems, which remains an underrepresented area in the literature. Still, there are very few studies taking place outside of the US, especially in Europe, Asia, Africa and South America. The first study using qualitative methods was a master’s thesis evaluating two interventions designed to reduce the feeding of deer by visitors in the US [26]. In the contemporary literature it is increasingly common to use qualitative methods to investigate behavioral change through interviews, fully thematic qualitative surveys or surveys including qualitative components [27]. Fewer studies investigate how the behavioral change affects natural habitat, plants, or animals. Only three of the studies do this [28–30] and we identified no apparent trend that attention to impacts to nature due to behavioral change is growing. The same applies for the use of experimental and quasi-experimental research design, three of these studies appeared in the 1990’s, four studies between 2000–2010 and three studies in recent years.

### Settings and behavior characteristics

We grouped the target behaviors within the 54 included studies into seven overarching behavior categories (Table 1). A total of eight studies examined multiple visitor behaviors within their case studies, resulting in a total of 68 categorized cases (e.g., a study that used the same communication strategy for both camping impacts and disposing waste). The primary behaviors targeted varied; 80% of the studies examined channeling use (type 1, e.g., hiking on designated trails), disposing waste (type 2, e.g., ‘pack in pack out’, picking up others’ trash or dispose waste in trash cans), respecting wildlife (type 3, e.g., do not feed or disturb wildlife while hiking or reef diving), or fee payment (type 4, e.g., giving donations) and regulation compliance (type 4, e.g., no illegal collection or harvest). Ten percent of behaviors targeted in the studies examined how to encourage visitors to act according to safety measures (e.g., staying away from high risk areas). The remaining studies examined a suite of behaviors related to minimizing camping and social impacts (e.g., tree damage, designated camping and/or campfire area use, or reduce noise), seven of which included proper waste disposal.

**Table 1:** The number of different behaviors targeted in studies investigating behavior change through communication measures in natural settings

	Channeling use (type 1)	Disposing waste (type 2)	Respect wildlife (type 3)	Fee payment and regulation compliance (type 4)	Safety (type 5)	Camping impacts (type 6)	Social impacts (type 7)	Total behaviors studied per country
US	13*	9*	5	8*	2	5*	1	43
Australia	1*	2*	5*	2*	2		1*	13
Japan	1*	1*	1*		1*			4
New Zealand					2			2
Canada	1							1
Argentina		1						1
Indonesia				1				1
Switzerland	1							1
Israel		1						1
Uganda		1						1
Total behavior type	17	15	11	11	7	5	2	68

^*^Included studies that examined multiple behaviors

Regarding the biophysical characteristics of the natural setting represented in visitor behavior studies, the primary settings were forest environments (41%, n = 22), followed by mountains and alpine regions (13%, n = 7), and woodland–grassland savannah biomes (9%, n = 5). Other settings included beaches, freshwater bodies (lakes and rivers), marine areas (coastal or reef areas), and municipal parks/urban green spaces. Sixty seven percent of the studies (n = 36) took place in protected areas, mostly national parks, while the remaining occurred in natural recreation settings. The behaviors targeted in protected areas were mostly channeling use (behavior type 1), disposing of waste (behavior type 2), and respecting wildlife (behavior type 3). Specifically, studies examined how to alter visitors’ behavior regarding crowding in space and time, keeping to maintained trail (behavior type 1), littering, surface disposal of human waste (behavior type 2), bird feeding, dogs off leash, keeping safe distance to wildlife, and food storage around bears (behavior type 3). The remaining behaviors concerned paying fees, comply with safety barriers, unsafe swimming, limiting the amount of noise generated by visitors, and limiting tree damage.

Despite a focus on visitor behavior and the use of communication to alter behavior, there is a paucity of information about the specificity of the *visitors* that the communication measures have targeted. For almost half of the studies the targeted population was not described in the published source (n = 23) and the focus was only or mostly on proportions of foreign and domestic visitors. Demographic descriptions such as age, gender, urban–rural, local–non-local and educational characteristics were conspicuously absent, despite a common focus on these characteristics in marketing and persuasion studies. Most studies stated that they had a mix of different visitors from locals to international visitors (32%, n = 17), but some had a focus on national visitors (24%, n = 13), other mostly on locals (9%, n = 5), or foreign visitors (6%, n = 3). Studies that targeted locals were primarily from the US (n = 4) and one from Australia. For those studies targeting only international visitors, these studies occurred in Uganda, New Zealand, and Indonesia. All studies that targeted international and/or national visitors occurred in national parks.

### Research design and methods used

Generally, studies testing the efficacy of visitor intervention via communication were observational in design (81%, n = 44). Ten studies were either quasi-experimental or fully experimental. The two studies that were fully experimental were virtual and/or game-based, in which they presented dilemmas to respondents they had to resolve with a particular behavior [31, 32]. The quasi-experimental studies typically examined waste disposal behavior where researchers would plant a study area with pieces of trash and observed number of trash items collected. Most studies had some sort of control, either pre- and post-test after implementing measure (n = 14, e.g., planting trash, clean up and tracking accumulation), or used a control group compared to a treated group (n = 35). Nearly one third of the studies (n = 16) used pre-tests in intercept surveys to examine the belief systems, attitudes, norms and perceptions of behavioral control of visitors in order to design communication interventions.

After interventions were launched, 39 studies used observation to measure behavioral change, mostly occurring as systematic moment observations in situ, but some (4 studies) with a camera trap. A majority (60% of all studies, n = 32) applied multiple methods to measure behavior change, such as observations combined with intercept surveys. A few studies (n = 5) did not include pre-/post-tests or control groups, because they typically examined communication measures already implemented at a site to explore the reasons for visitors’ non-compliance with communicated regulations/suggestions. In some cases, the baseline conditions at the study sites were communication measures already implemented without the guidance of communication theories, and the researchers examined behavioral effects and variation among visitor types. Examples in these baseline assessments include: an open-ended donation request with a blank space, a trash can without special signage, or other informational signs already present in the area. The ‘no-control’ studies and baseline assessment studies normally used intercept survey methods that included qualitative components or interviews to capture visitors’ own perception and researchers’ observations for reasons of behavioral change or for non-compliance. Other methods used in combination with observations and intercept surveys included: trail counters, big data (e.g., Strava), GPS trackers, passive surveys, assessments of impacts on nature, acoustical monitoring systems, voice recording, and tallying tickets, donations or waste.

The studies primarily utilized outcome measures that gathered quantitative data through observations, cameras, or trail counters to track changes in behavior and/or interviews and visitor surveys to explore reasons for non-compliance. The focus was primarily on documenting changes in visitor behavior (n = 54), with only a few studies (n = 3) also investigating the impact of communication interventions on the state of the natural resource before and after implementation. The studies were typically conducted over a brief time frame- usually during the peak tourist season- ranging from 3 days to 25 weeks. In addition, studies discussed norms and documented on-site contextual factors that may have affected behavior, such as visitation levels and landscape or infrastructure features.

### Communication measures studied

**Fig. 3:**
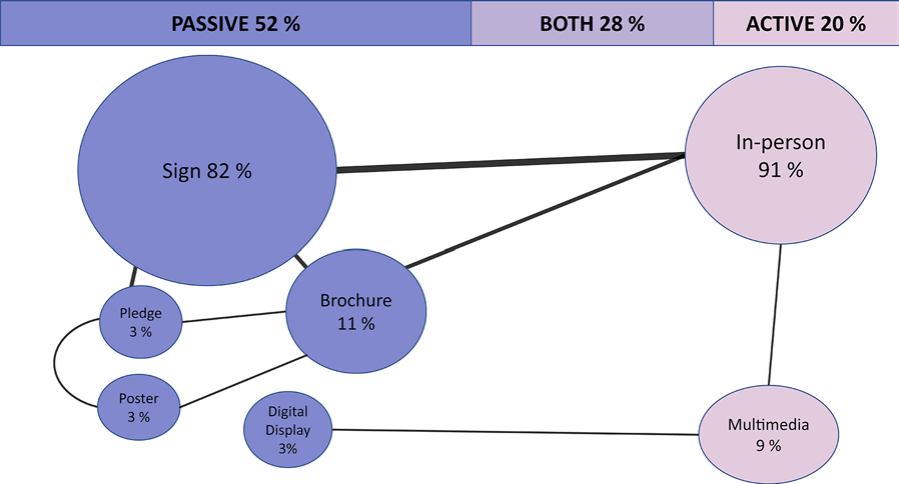
Bubble size illustrates the frequency of communication modes used to alter behavior in natural areas. The percentages are based on either the total passive (purple) or active (pink) messaging. The lines indicate that multiple measures (both, 28%) have been used in the same study: the thicker the line, the more frequent the combination

Most studies investigated only passive (written) messaging to alter visitor behavior (52%, n = 28), the most common being signs (n = 23) (Fig. 3). Passive messaging also included brochures (n = 3), pledges (n = 1), and posters (n = 1), sometimes used in combination with active messaging (n = 15, mostly personal contact). All told, 63% of the studies used signs in some way to try to change behavior (n = 34). From our included studies, we found 11 studies that only examined active messaging in situ about channeling use or waste behavior through in-person contact (one exception was multimedia targeting rule compliance). Regarding visitor type, active messaging was applied primarily in guided tours, but the passive messaging studies varied more in terms of studied behavior and applied communication measures.

### Theories and conceptual frameworks

Several theories were employed by researchers to design communication interventions, however a substantial part (37%, n = 20) of the studies did not clarify any specific communication or behavioral theory to guide their study at all. Of the theories applied, the theory of planned behavior dominated, and was used alone or in combination with other theories, such as the elaboration likelihood model (n = 18). These theories were, as stated by the authors, often combined aiming to increase the robustness of the design. Of similar popularity was norm theory (n = 9), followed by frame theory (n = 6) and moral foundation theory (n = 3). Other theories employed by researchers were identity theory, cognitive dissonance, extended parallel process model of fear appeals, assimilation-contrast theory, heuristic decision making, prosocial behavior theory, protection motivation theory, recreation demand hierarchy and attribution theory. Elements from the models of responsible environmental behavior and community-based marketing were also employed by a few studies.

**Fig. 4:**
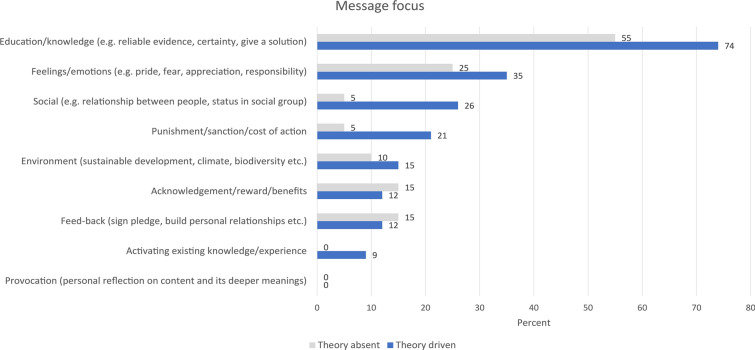
Different modes of messaging shown for studies employing communication/behavior

Most studies used several modes to deliver the message (63%, n = 34). Seven studies did not describe in sufficient detail the messages or the basis for them, so the coders placed the studies in broadly applicable themes. The most common theme was education and awareness raising (60%, n = 32, see Fig. 4). Feelings/emotions like pride, fear, appreciation and responsibility were also common themes when describing message framing (31%, n = 17). One fourth of the studies used social ‘incentives’ (e.g., relationship between people, status in social group, collective pride or shame) to activate behavioral change (n = 12). For studies using communication or behavior theories to develop the message it was common to use education, social norms and punishment/sanction or cause of action. These studies tended to use a higher number of different modes to deliver the messages than studies which did not use theories or frameworks to guide the communication process.

### Identified knowledge clusters and knowledge gaps

Our review identified four key knowledge clusters. First, we identified a knowledge cluster in intervening and measuring behavioral change to channeling use and handling waste disposal (food waste/storage and litter). Perhaps these topics have gained most research interest because they are easy to measure, monitor and test in the field using passive strategies, e.g., in situ observation of behavior changes by manipulation of signs and direct messaging. For example, testing the effectiveness of a sign on a trail that encourages the visitor to stay within a marked trail can lead to straightforward assessments of improvement or continued degradation. The map reveals a second area of knowledge that pertains to the use of outcome measures in studies, which focus on gathering quantitative data through observations, cameras, or trail counters to track changes in behavior. However, there is a limited amount of evidence on how behavioral change affects the state of the natural resource under consideration. Some authors raised concerns about whether behavioral change is long-lasting, but we argue that attention should also be paid to whether behavior change improves the state of the natural resource.

A third knowledge cluster we found in the literature is the use of passive communication to change behavior. This body of research underscores the significance of subtle adjustments in layout and wording since they can yield substantial differences in the effectiveness of persuasive messaging (see for example, [10]). While passive modes of communication are widely utilized, they often focus on simpler behaviors without employing behavioral theories to address more challenging behavioral changes. The amassed studies in this review tend to overlook that behavioral response may be a part of complex social context that is closely intertwined with political, cultural, legal, and economic issues (see e.g., [33]). In general, active communication tends to be more persuasive and impactful on personal behavior than mass-media(ted) communication (see e.g., [34]). However, we identified a fourth knowledge cluster that is primarily based on qualitative methodology, including data rich studies that examine existing communication strategies used by managers to assess effectiveness in behavioral change and explore the reasons for non-compliance. Studies characterized by this cluster explored the contextual reasons for observed behavioral change (or absence) including environmental, social and psychological aspects which provide invaluable evidence for furthering research in communication intervention design as tools for nature management.

Our review identified five key knowledge gaps. First and foremost, the mapping process showed that the literature is heavily skewed to measuring or predicting behavioral intentions, and rarely record and assess changes in actual behavior. The prevalence of behavior intentions in recreation research was reflected in our large pool of studies retrieved, but because the aim of the map was to examine the studies that included actual and observed behavior, our inclusion rate was less than 2%. From our assembled evidence we show that theory is often subsidiary to attempted behavioral change via communication, instead the studies often have a pragmatic approach studying how the context/setting contributes to behavioral change. For example, in waste studies, it was changing the context (littered vs. non littered settings) not the attitudes, norms, and perceptions of control—key elements in the widely used theory of planned behavior—which researchers prioritized to manipulate. This raises some concerns given the success of communication and value/attitude/norm-based theories in predicting behavioral intentions, but when actual behavior is tested—these same robust predictors play a minimal role or are absent from the study design. As a scholarly community, it behooves us to better understand how precedents to intended behavior differ from precedents to actual behavior, or if they do not, we should be better at explicitly using theoretical precedents to guide behavioral change measurements.

An important step researchers can take to improve the understanding of intentions and actual behavior relates to our second identified knowledge gap: conducting assessments of behavior across varied settings and visitor groups. As detailed in Fig. 2, we show that the geographical scope of the literature is in North American national parks and wilderness areas. The North America studies are not directly comparable to other part of the world. Theories of behavioral intentions in outdoor recreation in North America are rooted in specific management and governance structures, histories and traditions that are unique in some ways to these parts of the world. Furthermore, evidence suggests that people change their perceptions of their own behaviors (what is acceptable and what they actually perceive doing) while visiting a national park (see e.g., [35]). Such contextual factors have implications for how applicable theories of intended behavior apply to enacted behavior in parks as well as implications for how tenuous behavioral change may be from one setting to another.

A third knowledge gap is how long a behavioral change lasts. Here the literature is lacking in longitudinal assessments of behavioral change and supporting visitor perceptions on the new situation. We encourage conducting pre and post assessments of behavior over longer periods of time. A fourth knowledge gap is about effects of behavioral interventions by using both passive and active messaging. We identified a strong dominance of passive in situ messaging, in particular the use of signs. Future research can develop our knowledge of behavioral change by exploring effects of creative modes of both passive and active communication. The fifth key knowledge gap we identified is the paucity of typologies of the different visitor segments which the behavioral interventions are targeted towards. This is confounding as the communication and intended behavioral theories used to predict behavioral intentions often operate by having targeted population (sub-)groups. Behavioral intention theories are thereby built from pre-assessments of visitor types and other descriptive features. Observed behavioral change studies that test different measures in the field should include a clear definition of the different visitor types the study are targeting, in order to better understand behavioral changes for some groups but not all within the target population.

### Limitations of the map

We included literature written in English, and consequently, a part of the evidence base from other languages is not assessed. This could potentially address the major knowledge gap and biases we identified due to limited literature outside the North American visitor management tradition. Statistically significant results are more likely to be published than non-significant ones (negative results) and this can lead to overstating the positive effect of communication interventions [36]. Additionally, some studies that classified their study area as “protected area” appeared to deviate from the internationally IUCN categorization of protected area classes, thus should be interpreted with caution. Our analyses minimized biases in the search for articles, by including literature outside traditional academic bibliographic sources, by using multiple databases and also by including searches for older publications and grey literature [36].

## Conclusions

### Implication for policy/management

This systematic map contributes to the effort to promote sustainable outdoor recreation and nature-based tourism by providing information on communication measures used to change human behavior. The results from the review can be used as a starting point for detailed syntheses of the available evidence and an organized repository of relevant information for use in visitor- and conservation management.

Evidence was distributed unevenly among every study attribute we assessed: spatial scales, geographic scopes, environmental settings, behavior types, and measures used. The aquatic realm was the subject of substantially less research effort than the terrestrial realms. Geographically the abundance of studies was conducted in the US. In terms of target behaviors studied there was greater research attention on channeling visitors to designated areas or trails and to develop proper waste disposal. There was less focus on behaviors related to minimizing camping and social impacts. Similarly, some communication mediums were more frequently studied, with signs being the absolute mostly used, whilst other passive mediums were seldom studied e.g., pledge, poster, digital display, multimedia. Active messages trying to educate people by direct contact were used in some studies.

Much of the communication measures developed to support sustainable behavior aims to educate and increase awareness. The educational communication is based on the assumption that people do not act sustainably because they do not know how to act, or they do not know that their behavior is damaging the environment [6]. Eliminating knowledge gaps among the users through increased awareness and education will be a useful first step in supporting more sustainable behavior, but managers should consider other factors that may influence behavior. Studies have shown that visitors are more likely to engage in pro-environmental behaviors when messages leverage psychological factors such as identity, emotions and social influence [33, 37]. Our review shows that managers have a large pool of communication strategies and tools to use and adapt to their context and desired target behavior.

### Implications for further research

Our review documents that academic interest in how communication can increase sustainable behavior is growing. In line with Esfandiar et al. [15], we found that a substantial number of empirical studies of pro-environmental behaviors in natural areas hinged on cross-sectional quantitative surveys. Self-reported surveys may raise the issue of the social desirability bias of the responses and may not represent an individual’s actual behavior (i.e. unreliable results), which is why we focused on studies that measured observed behavior response. Our review supports earlier studies that have shown an inconsistency between reported behavioral intention and the behavior that were actually observed [38, 39]. Visitors often have strong intentions to act in a sustainable way, but that other intervening factors including those beyond their perception of control, have a stronger influence over their actual behavior. Existing theories have in specific domains succeeded in explaining behavior to a large extent, but the complexity of pro-environmental behaviors calls for a more comprehensive theory [40], including both qualitative and quantitative approaches. Scholars suggest to use different models in specific contexts and behaviors where they perform best [41] or combining existing theories into a framework that might describe all relevant factors influencing behavior [40].

We have identified a theory-intention-behavior gap**,** but this could arise due to difficulties in translating theoretical principles to practical, concrete, and highly context specific communication tools. As highlighted in our review, one third of the theory-driven studies applied the theory of planned behavior that is based on rational reasoning. An important critique to the rational approach is that the actual behavior is often guided by emotional and contextual factors. These include unconscious, associative and impulsive influences, affective associations, personal norms, habits, and self-identity [15, 39, 40, 42]. Most behavioral theories require specificity not only in the type of behavior under investigation, but also in situational aspects such as the targeted visitors, place and timing of the desired behavior [40, 43]. We advocate for testing and adapting new theories to better describe behavior complexity. For example COM-B model of behavior and the Behavior Change Wheel draw from a wide range of disciplines and approaches for designing and evaluating interventions [44]. We also advocate a broadening of qualitative research within the field to better understand the drivers for sustainable behavioral change in natural settings. A qualitative study could better define the potential drivers in the behavior phenomena studied.

We suggest conducting systematic reviews based on the evidence clusters identified in this map to enhance the understanding and effectiveness of communication measures to change behavior. Although some of the studies in this map used and compared different communication measures to target the same behavior, few explicitly compared different message framing on different visitor segments. Different visitor segmentation techniques should be investigated further to increase the precision of the research studies. We conclude that research should also investigate how the behavioral change actually alters the natural resource or environment in question, as behavioral change (both in adoption and non-compliance) can have unforeseen consequences to the state of the environment. Empirical comparisons of communication approaches are essential for progress, and we encourage researchers within the field to investigate this further. We need to know to what degree are different communication strategies effective, and in what contexts effective change occurs. In this line it is important to distinguish what aspects of behavior change could be generalized across settings, and which aspects are purely contextual that drive behavior change.

## Supplementary Information

**Additional file 1. **ROSES form for systematic map.

**Additional file 2.** Supplement A: Screening process. Supplement B: Eligibility criteria. Supplement C: Full text exclusion. Supplement D: Extracted data records.

## Data Availability

Data analyzed during this study is included in this published article and an overview and reference list of all data used are available in this article’s additional information files. The results from the systematic searches and article screening are also available in Sysrev (sofie.selvaag/On-site communication measures as a tool in outdoor recreation management).
